# Non-Infectious Choroiditis Subdivided into Its Diverse Pathophysiological Sub-Groups Is the Best-Known and Most Appropriate Nomenclature to Date for the Reclassification and Diagnosis of Former White Dot Entities

**DOI:** 10.3390/diagnostics16111735

**Published:** 2026-06-04

**Authors:** Carl P. Herbort Jr, Sukhum Silpa-archa, Ioannis Papasavvas, Nadia Bouchenaki, Anna Byszewska, Oleksandra Dorokhova, Christine Fardeau, Alireza Hedayatfar, De-Kuang Hwang, Vânia Lages, Wen-Jung Lo, Marina Papadia, Masaru Takeuchi, Yoshihiko Usui, Ryoji Yanai, Oleksandra Zborovska

**Affiliations:** 1 Inflammatory and Retinal Eye Diseases, Center for Ophthalmic Specialised Care (COS), 1003 Lausanne, Switzerland; 2 Department of Ophthalmology, Rajavithi Hospital, College of Medicine, Rangsit University, Bangkok 10500, Thailand; 3 Uveitis, Ophthalmology Practice Montchoisy, Rue de Montchoisy 5, 1207 Genève, Switzerland; nbouchenaki@proton.me; 4 Department of Ophthalmology, Military Institute of Medicine, National Research Institute, 01-045 Warsaw, Poland; abyszewska@wim.mil.pl; 5 Department of Inflammatory Pathology of the Eye, State Institution “The Filatov Institute of Eye Diseases and Tissue Therapy of the NAMS of Ukraine”, 65000 Odessa, Ukraine; dorochovaa@gmail.com (O.D.); oleksandra.v.zborovska@gmail.com (O.Z.); 6 Department of Ophthalmology, Reference Centre for Rare Diseases, Lariboisière Hospital, Assistance Publique-Hôpitaux de Paris, University of Paris Cité, 75014 Paris, France; christine.fardeau@aphp.fr; 7 Eye Research Center, The Five Senses Institute, Rassoul Akram Hospital, Iran University of Medical Sciences, Tehran 14535, Iran; alireza.hedayatfar@gmail.com; 8 Noor Ophthalmology Research Center, Noor Eye Hospital, Tehran 14535, Iran; 9 Department of Ophthalmology, Taipei Veterans General Hospital, Yilan County 264018, Taiwan; m95gbk@gmail.com; 10 School of Medicine, National Yang Ming Chiao Tung University, Taipei 112304, Taiwan; 11 Department of Ophthalmology, Unidade Local de Saúde do Alto Ave (ULSAAVE), 4835-044 Guimarães, Portugal; vania.mlages@gmail.com; 12 Department of Ophthalmology, Taichung Veterans General Hospital, Taichung 407219, Taiwan; kan42731@gmail.com; 13 Department of Ophthalmology, School of Medicine, National Yang Ming Chiao Tung University, Taipei 112304, Taiwan; 14 Program for Precision Health and Intelligent Medicine, Graduate School of Advanced Technology, National Taiwan University, Taipei 100225, Taiwan; 15 Department of Ophthalmology, Ospedale Evangelico Internazionale, 16100 Genova, Italy; marinapapadia@yahoo.com; 16 Department of Ophthalmology, National Defense Medical College, Tokorozawa 359-8513, Saitama, Japan; masatake@ndmc.ac.jp; 17 Department of Ophthalmology, Tokyo Medical University, Tokyo 160-0023, Japan; usuyoshi@gmail.com; 18 Department of Ophthalmology, Institute of Biomedical Sciences, Tokushima University Graduate School, Tokushima 770-8503, Japan; yanai@tokushima-u.ac.jp

**Keywords:** non-infectious choroiditis, choriocapillaritis, Primary Inflammatory Choriocapillaropathies (PICCPs), stromal choroiditis, Primary Inflammatory Stromal Choroiditis (PISC), Multiple Evanescent White Dot Syndrome (MEWDS), Acute Posterior Multifocal Placoid Pigment Epitheliopathy (APMPPE), Multifocal Choroiditis (MFC), serpiginous choroiditis, VOGT-Koyanagi-Harada disease (VKH), Birdshot Retinochoroiditis (BRC), Indocyanine Green Angiography (ICGA)

## Abstract

White dot syndromes (WDS), a purely descriptive term formulated in 1995, grouped inflammatory conditions primarily affecting the choroid that shared the clinical appearance of white dots on fundus examination, thus presenting similar white dots on fundus examination, which, however, had diverse pathophysiologies and different primary localizations of inflammation that did not warrant their association. Some of them, including birdshot retinochoroiditis (BRC) and sympathetic ophthalmia, produced predominant inflammation in the choroidal stroma, while others, including multiple evanescent white dot syndrome (MEWDS) and acute posterior multifocal placoid pigment epitheliopathy (APMPPE), caused inflammatory non-perfusion of the choriocapillaris. The aim of this review is to build upon a pathophysiology-based classification framework that groups these entities under the broader concept of non-infectious choroiditis and subdivides them according to their predominant inflammatory mechanisms and anatomical localization. This framework distinguishes diseases characterized by inflammatory occlusion of the choriocapillaris with consequent perfusion dysfunction, including MEWDS, APMPPE, multifocal choroiditis (MFC), and serpiginous choroiditis, from conditions primarily involving inflammatory infiltration of the choroidal stroma, such as birdshot retinochoroiditis (BRC), Vogt-Koyanagi-Harada disease (VKH), and sympathetic ophthalmia. Moreover, the review raises the question of how such an inadequate term, based on unscientific assumptions, could gain such quick access to ophthalmic practice and literature, be immediately adopted in major textbooks, and persist for so long being unrecognized as a misnomer. The slow and progressive reevaluation of this nomenclature over more than two decades is related and contextualized.

## 1. Introduction

The term white dot syndromes (WDS) was first proposed in 1995 to group choroidal disease entities that are not related to each other, based solely on a similar fundus appearance [1]. This inappropriate, nosologically imprecise, and nonsensical term rapidly and progressively infiltrated medical literature and practice. Although it was contested as early as 2002 [2,3,4,5,6], it was not until 2022 that a group of clinicians formally published a manifesto calling for the abandonment of this terminology [7]. It took them some time and two publications [8,9] to persuade an official uveitis society to abandon a newly planned meeting on WDS and instead transform it into a meeting aimed at replacing the term WDS. This was initiated by a questionnaire sent to clinicians at large by this group regarding their opinion of the “WDS” term, resulting in a very promising publication entitled “Is it time to adopt a new nomenclature and classification for white dot syndromes” [10]. After this first publication, 10 more publications followed; only a few are cited here [11,12,13,14,15,16]. These publications redefined the imaging parameters of the different former WDS entities, mostly already known and extensively described in the past, but nevertheless revisited by a core group with well-defined opinions. However, the flooding of the ophthalmic literature with these articles did not fulfill the task defined in the first article, namely, reclassifying WDS disease entities; instead, they simply described imaging features of the different diseases side by side without pathophysiological considerations, analysis, and classification, and without providing the announced new nomenclature. As in many consensus-driven processes, there is always the risk that the most influential experts will take the lead, with the rest tacitly following, resulting in conclusions potentially influenced by the composition and prevailing viewpoints of the expert panels involved.

The aims of the review were threefold: (1) to give a chronological overview of the rise and fall of the WDS terminology and its impact on ophthalmology practice through a literature search, (2) to reactivate an existing and well-defined nomenclature of these entities with a classification based on anatomopathological localization (choroidal stroma or choriocapillaris), and pathophysiological mechanisms, and finally (3) to highlight the importance of objective and balanced approaches in developing terminology and classifications that may support broadly applicable definitions and clinical guidelines.

## 2. The Rise and Fall of “White Dot Syndromes” (WDS)

**Figure 1:**
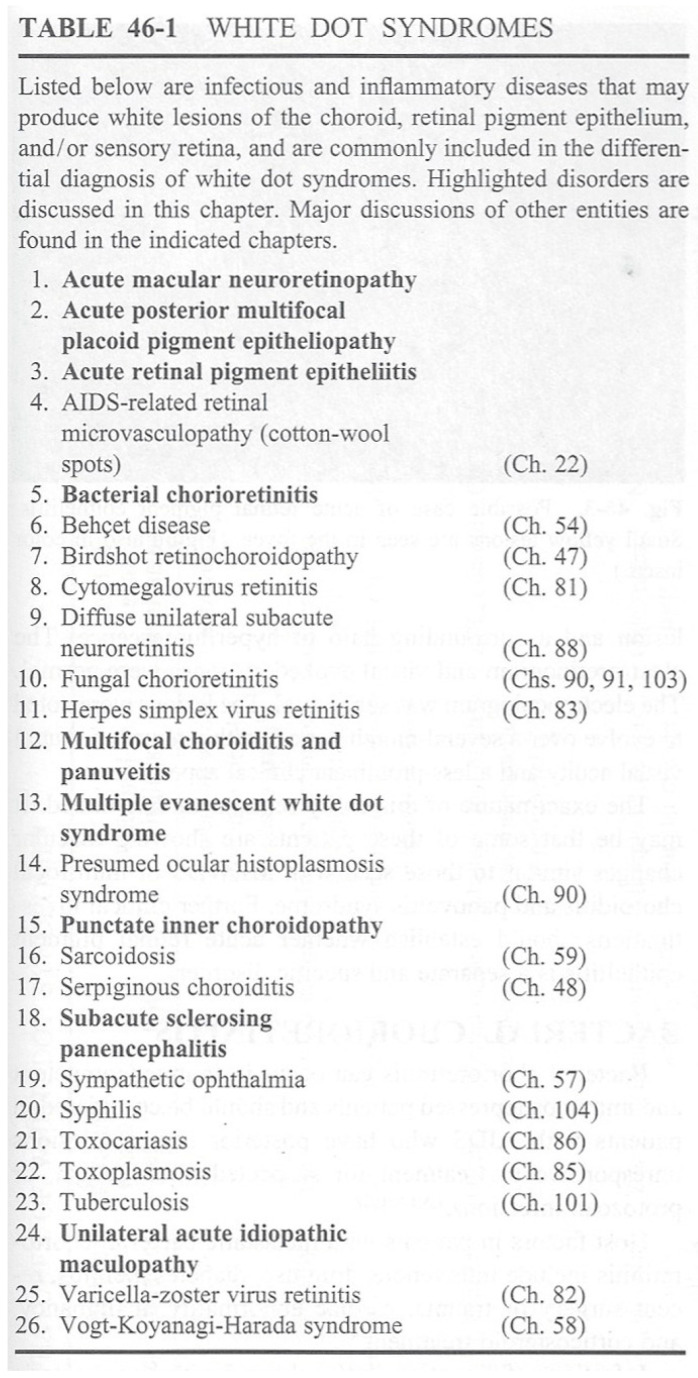
List of WDS as published in the book “Ocular Immunology and Infection” in 1996. Such a vast list simply corresponds to an enumeration of all white dot conditions without any qualifying purpose and has led to an overuse of the WDS term by further diluting its purpose, if there were any at all.

In 1995, an opinion article entitled “Fundal white dots: the spectrum of a similar pathological process” was published in the British Journal of Ophthalmology [1]. The article consisted of a mixture of assumptions and hypotheses, including clinical findings, histopathological data from granulomatous choroidal diseases such as sympathetic ophthalmia or sarcoidosis, and choroidal immunopathological conjectures, explaining that “fundal white dots are the clinical expression of the formation of microgranulomata”. One positive element that can be retained from this article is that the authors recognized that the location and primary development of the diseases they described were in the choroid. The diseases listed in this article include sympathetic ophthalmia, Vogt–Koyanagi–Harada disease (VKH), acute posterior multifocal placoid pigment epitheliopathy (APMPPE), birdshot retinochoroiditis (BRC), multiple evanescent white dot syndrome (MEWDS), punctate inner choroidopathy (PIC), discrete multifocal choroiditis, and diffuse unilateral subacute neuroretinitis. It is difficult to understand how such an article, entirely based on conjectures without a rigorous scientific approach, could have achieved such notoriety from one day to the next. This raises the question of the responsibility of opinion leaders. Indeed, the term "white dot syndrome" appeared in most uveitis textbooks as early as 1996, with a senseless list of 25 white dot syndromes in one of them (Figure 1).

**Figure 2:**
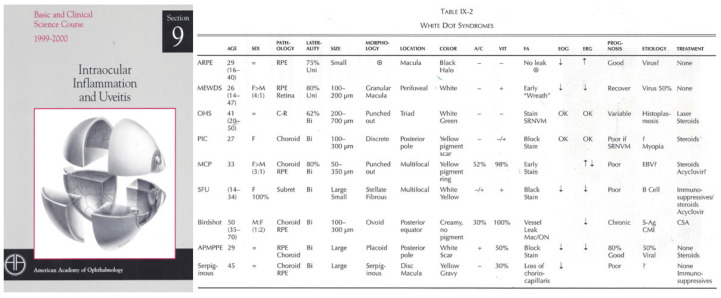
List of WDS in the Basic and Clinical Science Course 1999–2000 of the American Academy of Ophthalmology, Section 9 on Intraocular Inflammation and Uveitis. ARPE = Acute retinal pigment epithelitis, MEWDS = Multiple evanescent white dot syndrome, OHS = Ocular histoplasmosis syndrome, PIC = Punctate inner choroidopathy, MCP = Multifocal choroiditis and panuveitis syndrome, SFU = Subretinal fibrosis and uveitis syndrome, Birdshot, APMPPE = Acute posterior multifocal placoid pigment epitheliopathy, serpiginous.

Even the American Academy of Ophthalmology adopted the terminology in the uveitis section of its 1999–2000 edition of the Basic and Clinical Science Course and continued to use it until the latest edition (Figure 2).

As can be seen in Figure 2 and in the list presented in the 1995 white dots article, there was a regular evolution of entities and denominations within this group of diseases, some of which have since disappeared. Given its early adoption by authoritative sources, the WDS concept became widely disseminated in the literature, despite its unscientific and useless nature. Therefore, it was nearly impossible to go against such a mainstream trend for a long period of time. Indeed, once a notion is entrenched in the medical literature, it takes years, if not decades, to get rid of it, whatever alternative well-founded explanations, logical classifications, and terminology may be proposed. Therefore, beyond the phenomenological description of diseases, it is important to re-attract attention to the pathophysiology-derived nomenclature of former WDS entities, which was published some time ago and recalled more recently [17] in a presently mature era that is prone to abandoning this obsolete terminology, which is one of the purposes of this article.

What the authors of the 1995 article, however, intuitively understood was that the diseases they described primarily involved the choroid. Although indocyanine green angiography (ICGA) was already available at the time, it was not yet widely integrated into uveitis practice, and was not mentioned by them. Nevertheless, from 1993 to 1995, at least four publications on ICGA in uveitis were found in PubMe: three from Japan, a case report on MFC, and two case series on VKH [18,19,20]. In 1995, one center published the ICGA findings in a series of different chorioretinopathies [21]. The crucial progress brought by ICGA, emitting fluorescence in the infrared, was the fact that it enabled imaging of the choroid beyond the retinal pigment epithelium, which was not possible with FA. Because the choroid is primarily a vascular structure, ICGA represented substantial progress, as it made imaging of the choroidal circulation possible [22].

**Figure 3:**
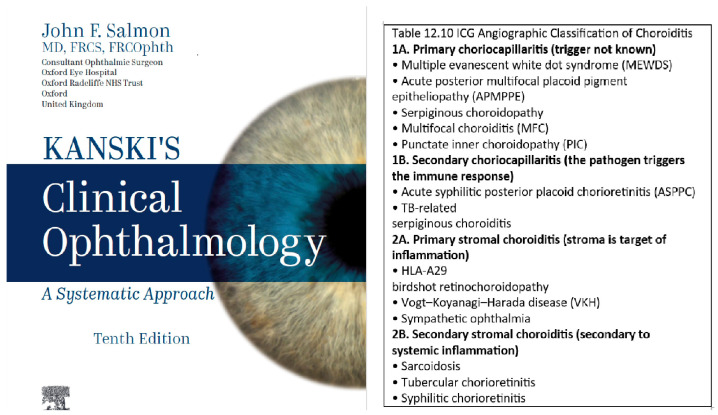
New nomenclature of non-infectious choroiditis in place of the white dot syndrome (WDS) terminology as proposed in the 10th edition of Kanski’s Clinical Ophthalmology.

With the help of ICGA, we reassessed these choroidal diseases since 2002 in several publications speaking of non-infectious choroiditis and proposing to subdivide them according to the vascular structure principally involved, whether the choriocapillaris or the stromal choroidal vessels [2,3,4,5,6]. Other groups maintained the name of WDS but subdivided it clearly into choriocapillaritis and stromal choroiditis [23]. Similarly, we renamed the diseases as non-infectious choroiditis and subdivided them according to localization and pathophysiology into choriocapillaritis and stromal choroiditis [17,24]. However, it was not until 2022 that an international group of clinicians from four continents published a manifesto clearly explaining the inadequacy of the term WDS and appealing for its abandonment [7]. This shift seems to have had a tangible impact in the field, as reflected by the 10th edition of Kanski’s Clinical Ophthalmology (2025), one of the most widely consulted references in ophthalmology, which replaced the WDS terminology with an ICGA-based classification of non-infectious choroiditis (Figure 3). The adoption of this framework in a major textbook may be viewed as an important milestone, indicating broader recognition of a pathophysiology-oriented approach within contemporary ophthalmic education and practice. This positive evolution in one of the leading ophthalmology textbooks has hopefully paved the way for the anticipated downfall of the inappropriate and useless WDS terminology, as the momentum seemed ripe for change.

## 3. Reclassifying White Dot Syndromes into Non-Infectious Choroiditis Subdivided into Its Diverse Pathophysiologic Framework

Choroidal inflammatory diseases could only be imperfectly investigated until ICGA became available in the early 1990s [18,19,20,21]. The absence or failure to use performing diagnostic methods to analyze choroidal structures led to misinterpretations of non-infectious choroiditis, such as the arbitrary grouping of these conditions under the white dot syndrome terminology.

### 3.1. Principles of ICGA Analysis of the Choroid

We would like to take this opportunity to reiterate the well-known basic principles of ICGA analysis of the normal and inflamed choroid. Although the current trend is to favor new non-invasive imaging methods such as enhanced-depth imaging OCT (EDI-OCT) or OCT angiography (OCTA), only ICGA is able to give global information on choroiditis to thus support the proposed classification.

#### 3.1.1. Normal Choroid

**Figure 4:**
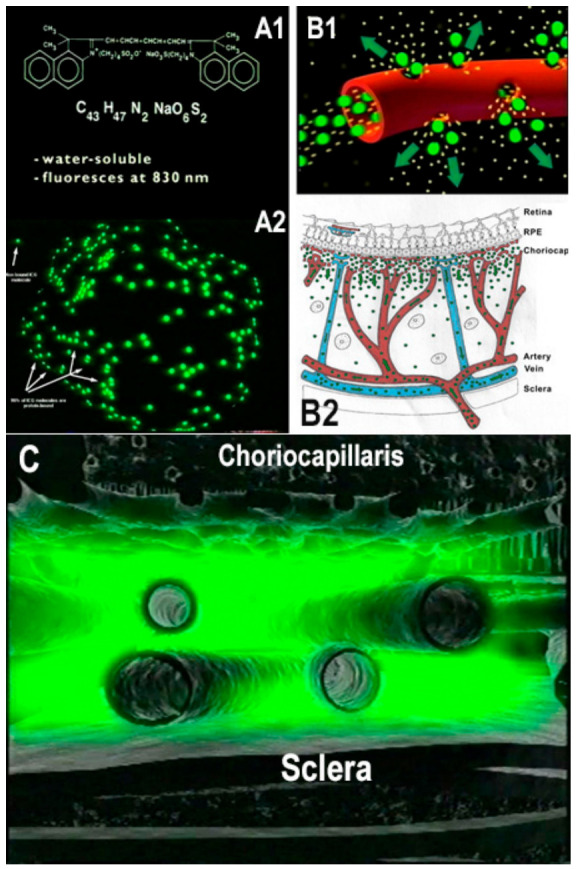
Principles of ICGA imaging of the choroid in a non-inflamed eye. The ICGA molecule is a small molecule of 775 Daltons (**A1**). However, 99% of ICG is bound to large proteins, forming a macromolecular complex of ±80,000 Daltons. The 1% of free ICG might be taken up by the RPE with a negligible impact on ICGA interpretation (**A2**). The macromolecular ICG-protein complex is freely extruding from the large fenestrations of the choriocapillaris (**B1**) and is progressively impregnating the choroidal stroma (**B2**). The large ICG-protein complex remains entrapped in the choroidal stroma because wash-out of this large molecular complex is slow (**C**).

The ICGA procedure showed that in a non-inflamed eye, the ICG dye, forming a large molecular complex composed of large proteins with attached ICG molecules, remained within large choroidal vessels but physiologically egressed from the large fenestrations of the choriocapillaris, progressively impregnating the choroidal stroma (Figure 4). Because of the large size of the protein-ICG complex, it remains trapped in the choroidal stroma, forming an ICGA-hyperfluorescent background, with faint ICGA fluorescence that can still be detected after 36–48 h post-injection. Potential uptake of this large 80,000-Dalton ICG-complex by the RPE plays a negligible role, if any, in late hyperfluorescence. In the non-inflamed eye, the background fluorescence is evenly distributed.

#### 3.1.2. Non-Infectious Choroiditis

**Figure 5:**
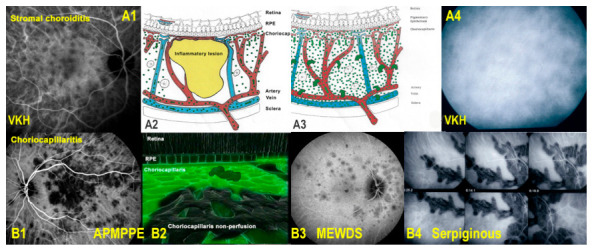
**ICGA-derived classification of non-infectious choroiditis (former WDS entities) into choriocapillaritis (pattern 1, bottom pictures) and stromal choroiditis (pattern 2, top pictures) based on location and pathophysiology of the lesion process.** Top pictures: example of stromal choroiditis (VKH) (**A1**) with round HDDs indicating absence of ICG dye because of space-occupying inflammatory foci illustrated by a cartoon (**A2**). In addition to HDD foci stromal choroiditis is accompanied by vasculitis of large choroidal vessels producing additional hyperfluorescence (**A4**) due to exudation from the usually impermeable large choroidal vessels as shown on cartoon (**A3**). Bottom pictures: examples of ICGA hypofluorescence due to inflammatory choriocapillaris hypo or non-perfusion as shown on the cartoon (**B2**) causing different degrees of hypofluorescent areas, depending on the size of vessels involved, pronounced in APMPPE (**B1**) with medium-sized vessels involved, very faint in MEWDS (**B3**) as small end-capillaries are involved and very pronounced in serpiginous (**B4**) where large choriocapillary or pre-choriocapillary vessels are involved..

In non-infectious choroiditis, the following main alterations of the ICG angiogram occur:

1. Choriocapillaris occlusion due to inflammation of small distal to large proximal choriocapillaris vessels, appearing as areas of hypofluorescence or absence of fluorescence due to the absence of dye in the non-perfused areas (pattern 1, choriocapillaritis) (Figure 5).
2. Choroidal stromal infiltration by inflammatory foci with concomitant vasculitis of larger stromal vessels, the space-occupying foci creating voids of dye (hypofluorescent dark dots = HDDs) and the vasculitic exudation producing additional fluorescence on top of the physiological fluorescence coming from the large fenestrations of the choriocapillaris(pattern 2, stromal choroiditis) (Figure 5).

Consequently, ICGA analysis of the former WDS entities, renamed as non-infectious choroiditis entities, allowed us to group these diseases into pattern 1 (choriocapillaritis) or pattern 2 (stromal choroiditis).

### 3.2. Choriocapillaritis

**Figure 6:**
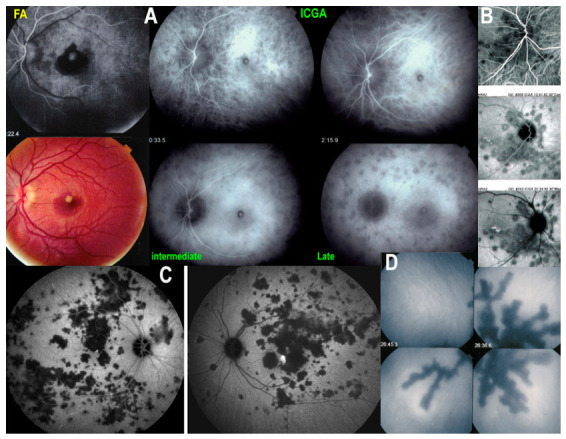
Examples of the main choriocapillaritis entities. Primary inflammatory choriocapillaropathies (PICCPs) (**A**) MEWDS is characterized by the least severe involvement of the choriocapillaris, involving small end-choriocapillaris vessel non-perfusion. (**A**) FA shows early choriocapillaris perfusion disturbance (**top left**). The ICGA quartet (**top center**) shows discrete early choriocapillaris hypoperfusion (33), best visible in the late angiographic phase. Secondary choroidal neovessels can complicate MEWDS, as in this case, a clear indication that the process is ischemic non-perfusion (**bottom left** of ICGA quartet). (**B**) Multifocal choroiditis (MFC) presents a similar pattern with denser hypofluorescent areas as larger choriocapillaris vessels are involved (top = early phase, middle = intermediate phase; lower frame = late phase. (**C**) APMPPE shows more pronounced choriocapillaris non-perfusion due to severe involvement of larger choriocapillaris vessels. (**D**) Serpiginous choroiditis is at the severe end of choriocapillaritis entities involving large choriocapillaris vessels or pre-choriocapillary vessels.

Choriocapillaritis entities are listed in Table 1 (adapted from Kanski’s Clinical Ophthalmology), showing the main diseases with pattern 1 (choriocapillaritis). In cases where the trigger of the disease is unknown, these entities are also called Primary Inflammatory Choriocapillaropathies (PICCPs). Comprehension of choriocapillaritis entities presupposes considering the anatomic location and severity of choriocapillaris inflammatory non-perfusion, involving small end-choriocapillaris vessels in MEWDS and benign MFC, with a progression in the severity of non-perfusion involving larger vessels in severe MFC and APMPPE, and very prominent and extensive non-perfusion of larger choriocapillaris vessels or precapillary vessels in serpiginous choroiditis (Figure 5 and Figure 6).

As for choriocapillaritis entities, in addition to the well-defined entities cited above, many intermediate forms have been reported, such as relentless placoid choroiditis and ampiginous choroiditis, among others, for which the etiology, similar to that of the main forms, has not been identified [25,26,27].

Furthermore, some cases of MFC and serpiginous choroiditis can be triggered by the tuberculous bacillus (positive interferon-gamma test) and are called TB-related MFC and/or serpiginous choroiditis [28,29]. Other cases of choriocapillaris non-perfusion can be caused by syphilis, producing acute syphilitic posterior placoid chorioretinitis (ASPPC) [30] (Table 1). Such cases, in which the trigger is known, are termed secondary choriocapillaritis or para-infectious choriocapillaritis. TB-serpiginous requires combined multiple anti-tuberculous and multiple immunosuppressive therapy. As for ASPPC, it has been shown that the process is immunologically mediated, as it can be suppressed by immunosuppressive therapy but recurs with discontinuation or decrease of therapy [30]. Indeed, the disease can only be eradicated by anti-treponemal antibiotic therapy.

**Table 1:** Classification of non-infectious choroiditis replacing WDS.

** 1. Choriocapillaritis** 1.1. Primary Inflammatory Choriocapillaritis Entities (PICCPs) (idiopathic, trigger not known) Multiple Evanescent White Dot Syndrome (MEWDS) (small terminal capillary occlusion → hypofluorescence, faint in early angiography before well pronounced on late frames) Multifocal choroiditis (MFC) (larger vessels involved → chorioretinal scars) Acute Posterior Multifocal Placoid Pigment Epitheliopathy (APMPPE) (Often larger vessels involved bilaterally in a single episode → large choriocapillary dropouts) Serpiginous Choroiditis (SC) (extensive and progressive bilateral large choriocapillary or pre-choriocapillary vessels involved → large serpentine scars) 1.2. Intermediary * and undefined ** choriocapillaritis entities Ampiginous choroiditis (Combination of APMPPE and SC features) Relentless Placoid Choroiditis (APMPPE features with recurrent evolution) Other non-classifiable choriocapillaritis entities (ICGA shows choriocapillaris non-perfusion, but cases cannot be classified into known phenotypes) 1.3. Secondary Choriocapillaritis Entities (trigger known) Tuberculosis-related Serpiginous Choroiditis (TB-serpiginous) (Interferon-gamma Release Assay/IGRA test positive) Acute Syphilitic Posterior Placoid Chorioretinitis (ASPPC) (Syphilis serology positive) * *Combines features of two phenotypically known entities* ** *Undefined forms correspond to choriocapillaritis cases on ICGA that cannot be classified into known phenotypes* **2. Stromal Choroiditis** 2.1. Primary Inflammatory Stromal Choroiditis (PISC) (Disease process arises from the choroid) Vogt–Koyanagi–Harada Disease (VKH) HLA–A29 Birdshot Retinochoroiditis (BRC) Sympathetic Ophthalmia (SO) 2.2. Secondary Stromal Choroiditis (The choroid is the innocent bystander and the chance location of a systemic disease) Sarcoidosis Chorioretinitis (non-exhaustive) Herpes-Zoster-related chorioretinitis

### 3.3. Stromal Choroiditis

Table 1 lists the main diseases with pattern 2 (stromal choroiditis), including VKH, BRC, and sympathetic ophthalmia. The analysis of these entities is more straightforward and has to be divided into primary forms, namely Primary Inflammatory Stromal Choroiditis (PISC), characterized by the fact that the lesion process directly originates from the choroid, which is the case for VKH, BRC and sympathetic ophthalmia, in contrast to sarcoidosis chorioretinitis, which results from a chance location in the choroid of a systemic disease and is therefore called secondary stromal choroiditis. Moreover, there is a growing body of literature on zoster-associated choroidopathy, particularly in recent years. Varicella zona choroidopathy can lead to large depigmented choroidal lesions, and the ICG may reveal underlying multifocal choroidal lesions [31].

## 4. The Importance of Unbiased Terminology and Disease Definitions to Achieve Universal Guidelines

The multimodal imaging in uveitis (MUV) Taskforce is a group of clinicians interested in uveitis, formed at a meeting organized by one of the uveitis societies. Initially, the meeting was going to be on the topic of WDS. In the course of the organization, the aim was, however, reoriented towards questioning the term “WDS” itself. Indeed, the publication in 2022 of an article by an international group calling for the abandonment of the WDS term reinforced the general trend towards abandoning the term [7]. Another strong incentive to orient the meeting towards redefining WDS was an opinion poll sent to clinicians interested in uveitis, in which 70% of participants advocated redefining the nomenclature and classification of WDS based on the primary anatomical location of the disease using multimodal imaging. Exactly such a classification based on anatomic location existed, as exposed in the introduction [2,3,4,5,6,7], and the process initiated by the MUV Taskforce raised hopes of confirming this result. The promising title of the article resulting from the opinion poll suggested such an evolution: “Is it time to adopt a new nomenclature and classification for white dot syndromes using multimodal imaging techniques” [10]. However, it has to be admitted that the MUV Taskforce, so far, has not fulfilled the objective defined in the title of their article and has not proposed the announced nomenclature [10].

Instead, the group redefined the imaging characteristics of each entity. Most of these diseases have been described for more than 40 years, APMPPE in 1968, Birdshot retinochoroiditis in 1980, MEWDS in 1984, and their imaging characteristics have been described throughout this period [24,32,33,34,35]. Nevertheless, the MUV Taskforce found it necessary to redefine them using a consensus-based technique, with the inherent risk of bias if such proceedings are not conducted rigorously, open to dissenting views, and report divergent points.

Another shortcoming of the MUV Task Force was that diseases were analyzed side-by-side, making them appear equivalent in terms of mechanisms, without the slightest hint of classifying them according to analytical criteria, including diverse pathophysiology and location, giving the impression that there were no structural/hierarchical differences among them.

We were interested in whether the approach of the MUV Taskforce was sufficiently unbiased to justify claims of universality. Therefore, we analyzed two conditions reported in their series of articles.

For instance, the MUV Taskforce’s interpretation of MEWDS emphasizes a single prevailing perspective, whereas a neutral appraisal would have required a balanced approach that also cited divergent positions regarding the primary photoreceptor disease theory. The asserted absence of early ICGA hypofluorescence, as put forward, cannot be accepted [35], and hence, the absence of choriocapillaris non-perfusion cannot be asserted [36,37,38,39]; many articles have left at least the question open [40]. Indeed, it is true that early ICGA hypofluorescence is faint in the early phase because terminal capillaries are involved with limited non-perfusion, in contrast to APMPPE, where larger vessels are involved with more extensive areas of capillary drop-out. The particular interpretation of MEWDS by the MUV Taskforce, as an outer retinal disease, while omitting mention of other equally plausible mechanisms, was also at the origin of discomfort about how to rename the group of former WDS diseases. As a consequence of considering MEWDS as a potentially non-choroidal entity, it became difficult to regroup the former WDS conditions under the specific and self-explanatory terminology of non-infectious choroiditis, but the MUV Taskforce had to adopt the broader designation of non-infectious posterior uveitis, which was inadequate to characterize the former WDS entities centered on choroidal inflammatory processes. Indeed, non-infectious posterior uveitis is a much more general term encompassing entities such as Behçet’s uveitis, autoimmune retinopathy, acute macular neuroretinopathy (AMN), unilateral acute idiopathic maculopathy (UAIM), Idiopathic Retinitis Vasculitis Aneurysms Neuroretinitis (IRVAN), retinal vasculitis, and many more that are not characterizing the choroid-centered former WDS diseases, fundamentally diverging in anatomical localization and/or pathophysiological mechanisms. Differentiation between OCT loss of photoreceptor outer segments due to primary photoreceptoritis on one side or to choriocapillaritis-induced loss on the other side is easily determined by ICGA, showing either non-perfusion (choriocapillaritis-induced) or absence of ICGA signs (photoreceptoritis) [41].

**Figure 7:**
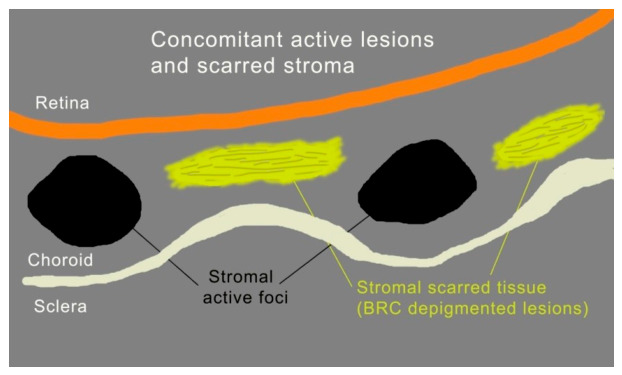
**Cartoon explaining the confusion about oval-shaped depigmented fundus lesions and hypofluorescent dark dots (HDDs) on ICGA.** Fundus lesions (yellow discs) are mid-stromal scars that have no expression on ICGA. Black round circles are active foci that prevent the diffusion of ICG and are seen as HDDs.

The appraisal of birdshot retinochoroiditis given by the MUV Taskforce also appears somewhat distorted, with several inadequacies. For this publication, a subcommittee of knowledgeable uveitis specialists presided over the choice of criteria on imaging and diagnosis that were then presented for vote, a process that appeared to us prone to biases, as all experts came from the same uveitis society, strongly influenced in this process by a well-known uveitis standardization group, while inclusion of a wider range of perspectives from outside the group could have enriched the process and supported the broader applicability of the proposed parameters. Moreover, analysis of a disease cannot rely on only 15 cases, a methodological inadequacy. If the points presented by the sub-committee were inadequate, the process could not be validated, as already mentioned, by the fact that there was a general vote. One hint that preferences were operating in the process is the fact that relevant publications from outside the MUV Taskforce were omitted, including articles on the specificities of retinal involvement in BRC [42,43,44]. Under the paragraph “Fundus Fluorescein Angiography”, it is said that the study cohort did not include cases with clinically apparent disc oedema, a very questionable choice. It is clear and recognized today that disc oedema is a prominent feature of BRC. Indeed, at least two studies reported that 100% of birdshot cases showed profuse FA disc oedema [43,44]. The MUV Taskforce article correctly mentioned that ICGA can show that “chorioretinal (the authors probably meant choroidal) birdshot lesions may completely disappear without residual scarring” following treatment. However, they failed to cite the article that described this feature [45] and cited instead, probably not purposefully, an unrelated paper speaking of FA disc oedema in BRC [43]. Another inadequacy that would have been avoided if an outside review had been sought is that the fundus autofluorescence (FAF) image in Figure 2 shows the left eye, which does not match the fundus, FA, and ICGA images showing the right eye. MUV Taskforce Report No. 8 was not clear about the relation between clinical fundus lesions and ICGA hypofluorescent dark dots (HDDs). The report says under the heading “Indocyanine Green Angiography” that “hypofluorescent choroidal lesions often extend beyond what is clinically visible”, implying that ICGA is showing fundus depigmented birdshot lesions, which is incorrect. Indeed, this wording could be interpreted as suggesting correspondence between HDDs and fundoscopically visible depigmented birdshot lesions. Current understanding indicates that classical birdshot fundus depigmented lesions are scars in the mid-stroma and do not appear on ICGA. Moreover, in the early phase of the disease without RPE damage, none of the active round HDDs seen on ICGA are visible funduscopically or on FA. Clarifying these distinctions would have improved the understanding of imaging findings and their relation to disease activity [46] (Figure 7). Only full-thickness atrophic areas in very advanced disease appear as protean hypofluorescence. Classical round ICGA HDDs always correspond to active lesions and are the best biomarkers of global disease activity, as was convincingly shown by Cao et al. in C Stephen Foster’s group, whose positions in uveitis usually rely on an evidence-based approach independent from consensus-type groups [47]. To sum up, the incomplete and sometimes erroneous BRC characteristics presented by the MUV Taskforce Report No. 8 give a partial and inadequate view of BRC. An outside input or review before publication would have allowed a more balanced presentation. For the clinician not familiar with BRC, it is probably more useful to consult several viewpoints to have more diverse and comprehensive information that might be encountered in clinical situations, rather than having fixed and unilateral results coming from a limited group not including divergent views.

## 5. Conclusions

Reclassification of WDS entities has been proposed for more than 20 years [2,3,4,5,6], but after the incomprehensible enthusiasm for this completely inadequate term, the time was not ripe in the past, and this gives an idea of the meanderings of medical thought. The need to dismiss WDS has gained momentum since 2022, when an international group of clinicians recommended abandoning the WDS terminology to logically replace the name of this group of choroidal diseases with the term non-infectious choroiditis, subdivided into choriocapillaritis and stromal choroiditis according to pathophysiological considerations [7]. This new nomenclature was recently quasi-officially adopted when it was introduced in the 10th edition of one of the leading ophthalmology textbooks [48]. Additionally, in 2025, one of the uveitis societies also attempted to deconstruct the WDS terminology, nevertheless failing to ”Adopt a New Nomenclature and Classification for White Dot Syndromes” [10]. It should be noted that this process had already been completed in the multi-authored article published in 2022 [7], as well as in previously published articles by our group and other groups [17,23,49]. The activity of the MUV Taskforce instead has largely focused on redescribing already well-established existing imaging parameters in a series of approximately 10 articles published without outside review, passing these entities through the mill of closed consensus decisions, and putting forward a particular way of thinking in the field.

In this context, we propose to reassert a simple, evidence-based approach to the question through a self-explanatory system that is easy to understand for the non-specialized clinician in uveitis and relies on sound pathophysiological arguments.

Regardless of differing interpretative perspectives, what matters is dismissing an inappropriate and useless concept that prevailed for 30 years and restoring a logical, evidence-based, and easy-to-understand nomenclature for these diseases.

## Data Availability

No new data were created or analyzed in this study.

## Abbreviations

The following abbreviations are used in this manuscript:

WDS	White dot syndromes
PICCPs	Primary Inflammatory Choriocapillaropathies
PISC	Primary Inflammatory Stromal Choroiditis
MEWDS	Multiple Evanescent White Dot Syndrome
APMPPE	Acute Posterior Multifocal Placoid Pigment Epitheliopathy
MFC	Multi-focal Choroiditis
PIC	Punctate inner choroidopathy
TB-serpiginous	Tuberculosis-related Serpiginous Choroiditis
VKH	Vogt–Koyanagi–Harada disease
SO	Sympathetic Ophthalmia
MUV	Multimodal imaging in uveitis
BRC	Birdshot Retinochoroiditis
ICGA	Indocyanine Green Angiography
AMN	Acute macular neuroretinopathy
UAIM	Unilateral acute idiopathic maculopathy
IRVAN	Idiopathic Retinitis Vasculitis Aneurysms Neuroretinitis
ASPPC	Acute syphilitic posterior placoid chorioretinitis
HDDs	Hypofluorescent dark dots
OCT	Optical coherence tomography
EDI-OCT	Enhanced depth imaging OCT
